# Uncovering the genomic potential of the Amazon River microbiome to degrade rainforest organic matter

**DOI:** 10.1186/s40168-020-00930-w

**Published:** 2020-10-30

**Authors:** Célio Dias Santos-Júnior, Hugo Sarmento, Fernando Pellon de Miranda, Flávio Henrique-Silva, Ramiro Logares

**Affiliations:** 1 Molecular Biology Laboratory, Department of Genetics and Evolution – DGE, Universidade Federal de São Carlos – UFSCar, Rod. Washington Luis KM 235 - Monjolinho, São Carlos, SP 13565-905 Brazil; 2 Institute of Science and Technology for Brain-Inspired Intelligence – ISTBI, Fudan University, Handan Rd 220, Wu Jiao Chang, Yangpu, Shanghai, 200433 China; 3 Laboratory of Microbial Processes & Biodiversity, Department of Hydrobiology – DHB, Universidade Federal de São Carlos – UFSCar, Via Washington Luis KM 235 - Monjolinho, São Carlos, SP 13565-905 Brazil; 4 Centro de Pesquisas e Desenvolvimento Leopoldo Américo Miguez de Mello, Petróleo Brasileiro S.A. (Petrobras), Av. Horácio Macedo 950, Rio de Janeiro, RJ 21941-915 Brazil; 5 Institute of Marine Sciences (ICM), CSIC, Passeig Marítim de la Barceloneta 37-49, ES08003, Barcelona, Catalonia Spain

**Keywords:** Amazon River, Freshwater bacteria, Biodiversity, Metagenomics, Lignin degradation, Cellulose degradation, Priming effect, Gene catalogue

## Abstract

**Background:**

The Amazon River is one of the largest in the world and receives huge amounts of terrestrial organic matter (TeOM) from the surrounding rainforest. Despite this TeOM is typically recalcitrant (i.e. resistant to degradation), only a small fraction of it reaches the ocean, pointing to a substantial TeOM degradation by the river microbiome. Yet, microbial genes involved in TeOM degradation in the Amazon River were barely known. Here, we examined the Amazon River microbiome by analysing 106 metagenomes from 30 sampling points distributed along the river.

**Results:**

We constructed the *Amazon River basin Microbial non-redundant Gene Catalogue* (AMnrGC) that includes ~ 3.7 million non-redundant genes, affiliating mostly to bacteria. We found that the Amazon River microbiome contains a substantial gene-novelty compared to other relevant known environments (rivers and rainforest soil). Genes encoding for proteins potentially involved in lignin degradation pathways were correlated to tripartite tricarboxylates transporters and hemicellulose degradation machinery, pointing to a possible *priming effect*. Based on this, we propose a model on how the degradation of recalcitrant TeOM could be modulated by labile compounds in the Amazon River waters. Our results also suggest changes of the microbial community and its genomic potential along the river course.

**Conclusions:**

Our work contributes to expand significantly our comprehension of the world’s largest river microbiome and its potential metabolism related to TeOM degradation. Furthermore, the produced gene catalogue (AMnrGC) represents an important resource for future research in tropical rivers.

Video abstract.

## Background

Continental waters play a major biogeochemical role by linking terrestrial and marine ecosystems [1]. In particular, rainforest rivers receive large amounts of terrestrial organic matter (TeOM), which may then reach the ocean. TeOM is difficult to degrade (i.e. recalcitrant), being normally processed in rivers by microorganisms, stimulating its conversion to carbon dioxide [2–4]. Therefore, riverine microbiomes should have evolved metabolisms capable of degrading TeOM. Even though the gene repertoire of river microbiomes can provide crucial insights to understand the links between terrestrial and marine ecosystems, as well as the fate of organic matter synthesized on land, very little is known about the genomic machinery of riverine microbes that degrade TeOM.

Microbiome gene catalogues allow the characterization of functional repertoires, linking genes with ecological function and ecosystem services. Recently, large gene catalogues have been produced for the global ocean [5–7], soils [8] and animal guts [9, 10]. In particular, ~ 47 million genes have been reported for the global ocean microbiome [11] and ~ 160 million genes for the global topsoil microbiome [8]. Although functional metagenomics was already performed in the Amazon River [12–18], so far, no comprehensive gene catalogue was generated, which hinders our understanding of the genomic machinery that degrades almost half of the 1.9 Pg C discharged into rivers every year as recalcitrant TeOM [1]. This is particularly relevant in tropical rainforests, like the Amazon forest, which accounts for ~ 10% of the global primary production, fixing 8.5 Pg C per year [19, 20]. The Amazon River basin comprises almost 38% of continental South America [21], and its discharge accounts for 18% of the world’s inland-water inputs to the oceans [22]. Despite its relevance for global-scale processes, there is a limited understanding of the Amazon River microbiome.

Large amounts of organic and inorganic particulate material [23] turn the Amazon River into a turbid system. High turbidity reduces light penetration, and consequently, the Amazon River has very low rates of phytoplankton production [24], meaning that TeOM is the major carbon source for microbial growth [25]. High respiration rates in Amazon River waters generate a CO_2_ super-saturation that leads to its outgassing to the atmosphere. Overall, Amazon River outgassing accounts for 0.5 Pg C per year to the atmosphere [26], almost equivalent to the amount of carbon sequestered by the forest [19, 20]. Despite the predominantly recalcitrant nature of the TeOM that is discharged into the Amazon River, heterotrophic microbes are able to degrade up to ~ 55% of the lignin produced by the rainforest [27, 28]. The unexpectedly high degradation rates of some TeOM compounds in the river was recently explained by the availability of labile compounds that promote the degradation of recalcitrant counterparts, a mechanism known as *priming effect*, which has been observed in incubation experiments [28].

Determining the repertoire of gene functions in the Amazon River microbiome is one of the key steps to understand the mechanisms involved in the degradation of complex TeOM produced in the rainforest. Given that most TeOM present in the Amazon River is lignin and cellulose [27–31], the functions associated with their degradation were expected to be widespread in the Amazon microbiome. Instead, these functions exhibited very low abundances [16, 17, 32], highlighting our limited understanding of the enzymes involved in the degradation of lignin and cellulose in aquatic systems.

Cellulolytic bacteria use an arsenal of enzymes with synergistic and complementary activities to degrade cellulose. For example, glycosyl hydrolases (GHs) catalyse the hydrolysis of glycoside linkages, while polysaccharide esterases support the action of GHs over hemicelluloses and polysaccharide lyases promote depolymerization [33, 34]. In contrast, lignin is more resistant to degradation [35, 36], since its role is preventing microbial enzymes from degrading labile cell-wall polysaccharides [37]. The microbial production of extracellular hydrogen peroxide, a highly reactive compound, is the first step of lignin oxidation mediated by enzymes, like lignin peroxidase, manganese-dependent peroxidase and copper-dependent laccases [33]. Lignin oxidation also produces a complex mixture of aromatic compounds, which compose the humic fraction of dissolved carbon detected in previous studies in the Amazon River [29, 30]. Our knowledge of bacterial-mediated lignin degradation in the Amazon River is limited; however, it is known that in tropical streams bacterial lignocellulose degradation tends to occur in the entire water column, being slow and also predominantly modulated by bacteria in anoxic regions close to sediments [38–41].

Here, we produced the first gene catalogue of the world’s largest rainforest river by analysing 106 metagenomes (~ 500 × 10^9^ base pairs), originating from 30 sampling points covering a total of ~ 2106 km, from the upper Solimões River to the Amazon River plume in the Atlantic Ocean. This gene catalogue was used to examine the genomic machinery of the Amazon River microbiome potentially responsible for metabolizing large amounts of organic carbon originating from the surrounding rainforest. Specifically, we ask: How novel is the gene repertoire of the Amazon River microbiome? Which are the main functions potentially associated with TeOM degradation? Do TeOM degradation-related genes and functions display a spatial distribution pattern? And finally, is there any evidence of *priming effect* in TeOM degradation?

## Results

### Cataloguing the genes of the Amazon River microbiome

**Fig. 1:**
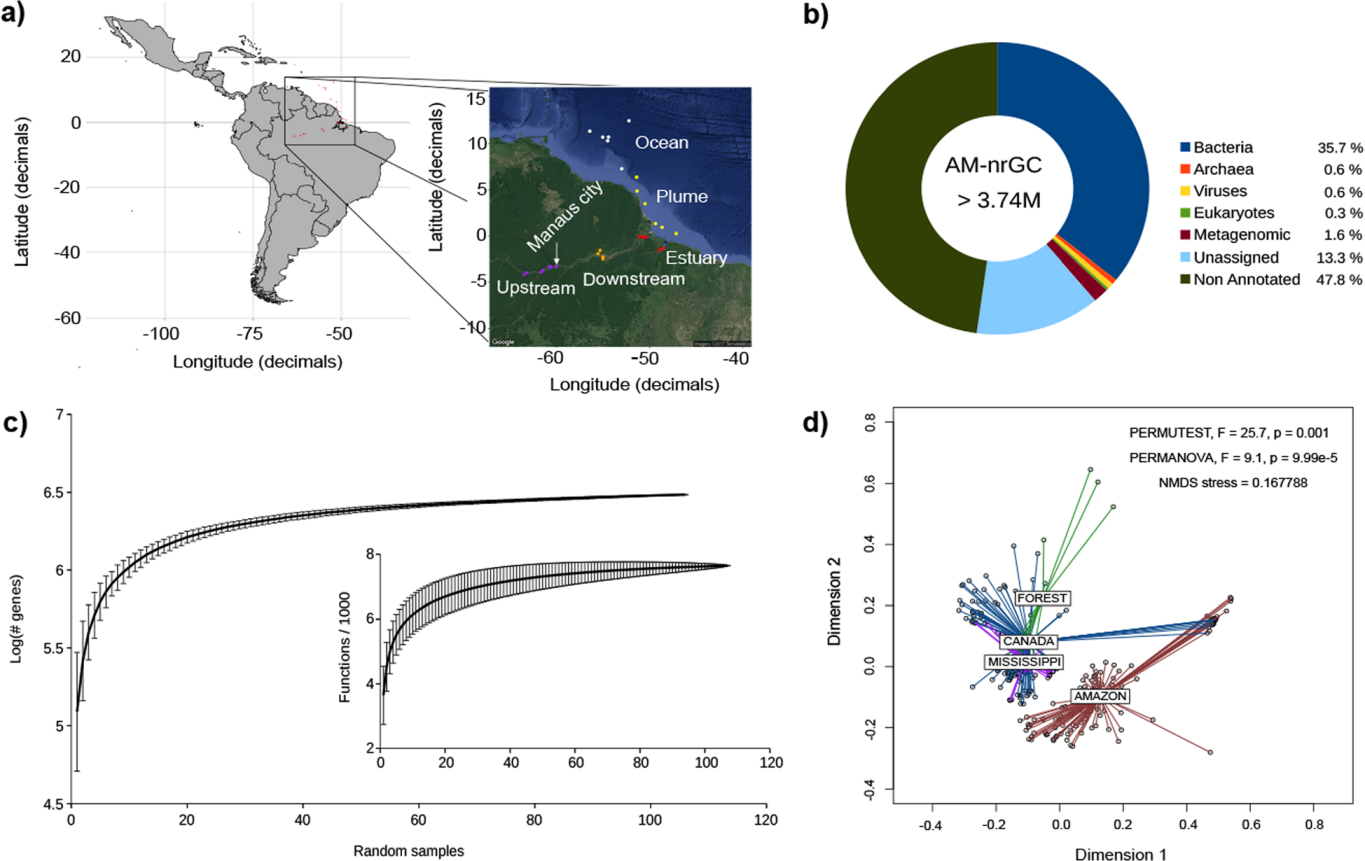
The Amazon River basin Microbial Non-Redundant Gene Catalogue (AMnrGC). **a** Distribution of the 106 metagenomes used in this work over the five sections of the Amazon River: Upstream (purple dots), Downstream (orange dots), Estuary (red dots), Plume (yellow dots) and coastal Ocean (white dots). **b** Taxonomic classification of the ~ 3.7 million genes in the AMnrGC. “Unassigned” genes were not assigned taxonomy, but they were functionally assigned, differently from “non-annotated” genes, which do not have any ortholog. Those genes displaying orthology to poorly characterized genes found in metagenomes were referred to as “Metagenomic”. **c** Rarefaction curves of non-redundant genes and PFAM families (internal plot). Note both point towards saturation. **d** NMDS comparing the Amazon river microbiome with other microbiomes based on information content [k-mer composition]. Amazon River (AMAZON), Amazon forest soil (FOREST), Canada watersheds (CANADA) and Mississippi River (MISSISSIPPI)

Amazon River genes were predicted after co-assembling 106 metagenomes (Supplementary Tables 1 and 2 in Additional file 1) in groups that shared the same geographic origin (Fig. 1a). We predicted 6,074,767 genes longer than 150 bp, allowing for alternative initiation codons. After redundancy removal by clustering genes with an identity > 95% and an overlap > 90% of the shorter gene, the *Amazon River basin Microbial non-redundant Gene Catalogue* (AMnrGC) included 3,748,772 non-redundant genes, with half of the genes with a length ≥ 867 bp (publicly available in Zenodo, doi: 10.5281/zenodo.1484504). About 52% of the AMnrGC genes were annotated with at least one database, while ~ 86% of the annotated genes were simultaneously annotated using two or more different databases. The recovered gene and functional diversity seemed to be representative of this microbiota as indicated by the leveling off of the rarefaction curves of genes and functions (Fig. 1c).

### The Amazon River microbiome differed from other microbiomes

We compared the metagenomic information contained in the Amazon River microbiome with that from the Amazon rainforest soil and other available temperate rivers (Canada watersheds and Mississippi River) using k-mers (Supplementary Table 3 in Additional file 1). The k-mer diversity comparison of these microbiomes indicated that they are different in terms of genomic composition (Fig. 1d), forming groups of heterogeneous constitution (significant *β* dispersion [that is, average distance of samples to the group centroid]—PERMUTEST, *F* = 25.7, *p* < 0.001). In particular, the k-mer composition of Amazon River samples was markedly different to the other microbiomes (PERMANOVA, *R*^2^ = 0.10, *p* = 9.99 × 10^−5^; ANOSIM, *R* = 0.27, *p* < 0.001), which suggests that this basin, or tropical rainforest rivers in general, may contain specific gene repertoires.

**Fig. 2:**
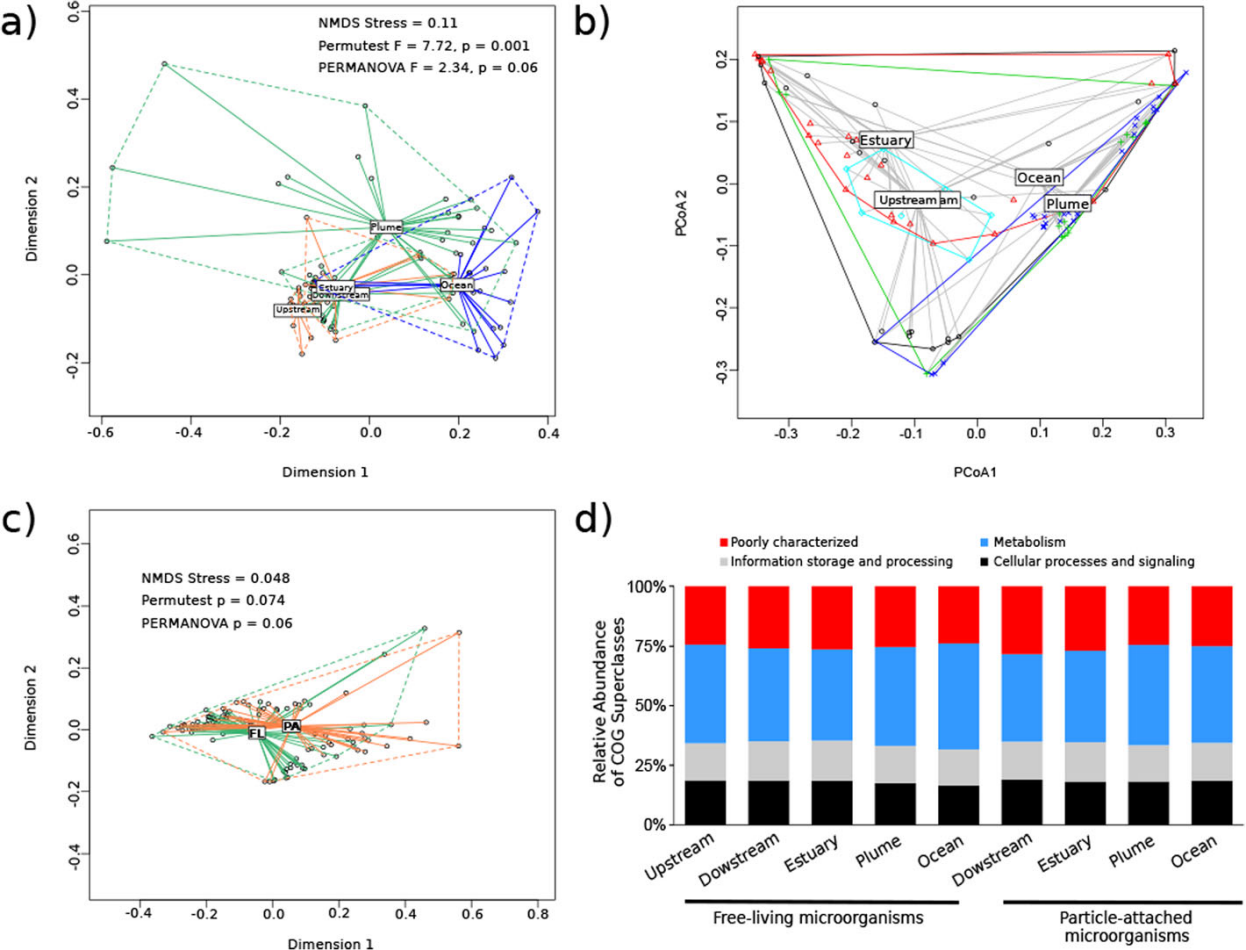
Metagenomic and COG composition of the studied sections of the Amazon River microbiome. Ordination of metagenomes composing the different river sections based on the Jaccard distances calculated from the presence-absence of k-mers in each sample (**a-c**). NMDS groups were statistically different [PERMANOVA, *F* = 2.34, *p* value = 9.99e−5] (**a**), displaying intragroup heterogeneity [*β* dispersion; PERMUTEST, *F* = 7.72, *p* value = 0.001] (**b**). Metagenomic composition of the Amazon River microbiome according to microbial lifestyle (free-living (FL) vs. particle-attached (PA)) (**c**). NMDS groups were statistically different [PERMANOVA, *F* = 3.62, *p* value = 0.06], displaying intragroup homogeneity [*β* dispersion; PERMUTEST, *F* = 3.62, *p* value = 0.074]. COG composition across size fractions and sections of the Amazon River (**d**). Gene functions grouped into COG superclasses are shown per river section and microbial lifestyle (free-living vs. particle-attached). The Upstream river section is not shown in the particle-attached fraction since it was not sampled

The metagenomic composition (k-mer based) of the five sampled sections of the Amazon River (i.e. upstream, downstream, estuary, plume and ocean) displayed significant differences (PERMANOVA test, *F* = 2.34, *p* < 9.9e−5; Fig. 2a), indicating that river sections may include different gene assemblages. These groups representing river sections were considered heterogeneous, as there was a significant *β* dispersion (*F* = 7.7, *p* = 1e−3) among metagenomic samples in each group (Fig. 2b). Additionally, the freshwater samples from different river sections (upstream, downstream and estuary) had shorter distances to centroids than those of brackish and marine samples (Fig. 2b). Even though we have used different size fractions to capture free-living or particle-attached microbes, this did not influence the k-mer composition (PERMANOVA test, *F* = 3.62, *p* = 0.06; *β* dispersion, *F* = 3.62, *p* = 0.074; Fig. 2c).

### Gene identification

About 48% of the AMnrGC genes could not be annotated due to lack of orthologs in reference databases. Besides, even though ~ 1.6% of the genes in the AMnrGC were previously found in metagenomic studies, they were poorly characterized, without being assigned to a particular taxon (here referred to as “Metagenomic” genes; Fig. 1b). Genes annotated exclusively through hidden Markov models (HMM) represented 13.3% of the AMnrGC. As the annotation using HMM profiles does not rely on direct orthology to specific sequences, but on orthology to a protein family (which may include mixed taxonomic signal), we could not assign taxonomy to those genes and they are referred to as “Unassigned genes” (Fig. 1b).

Overall, the previous results highlight our limited understanding about the gene composition of the Amazon River microbiome, where most proteins (61.1%) do not have orthologs in main reference databases. Prokaryotic genes (35.7% bacterial and 0.6% archaeal) constituted the majority of the AMnrGC, with only 0.3% and 0.6% of the genes having eukaryotic or viral origin, respectively (Fig. 1b).

### Core metabolisms

Functional analysis comprised prokaryotic and eukaryotic genes matching COG, KEGG and/or PFAM databases. The superclass “Metabolic processes” from the Clusters of Orthologous Genes (COG) database comprises those gene functions belonging either to energy production and conversion, amino acids, nucleotides, carbohydrates, coenzymes, lipids and inorganic ions transport and metabolism, secondary metabolites biosynthesis, transport and catabolism. This superclass was the most abundant in the AMnrGC (35.8% of the genes annotated with COG; Fig. 2d). Genes with unknown function represented 21.4% of the COG annotated proteins. Functionally, microbial lifestyle (i.e. free-living vs. particle-attached) did not influence the COG superclass distribution (Fig. 2d).

Core metabolic functions are those involved in cell or ecosystem homeostasis, normally representing the minimal metabolic machinery needed to survive in a given environment. KEGG and PFAM databases were used to determine the bacterial functional core, allowing also the identification of metabolic pathways. Core functions represented ~ 8% of KEGG and PFAM functions and were mostly related to general carbon metabolism, being predominantly associated with organic matter oxidation to CO_2_ and respiration byproducts heading to acetogenic pathways. Apart from core metabolisms, abundant proteins can reveal essential biochemical pathways in microbiomes. The top 100 most abundant functions in the bacterial core were “house-keeping” functions involved in main metabolic pathways (e.g. carbohydrate metabolism, *quorum* sensing, transporters and amino acid metabolism), as well as important protein complexes (e.g. RNA and DNA polymerases and ATP synthase). The non-core metabolism suggests adaptations to a complex environment, including multiple genes related to xenobiotic biodegradation and secondary metabolism (that is, the production and consumption of compounds not directly related to cell survival).

### The potential TeOM degradation machinery

**Fig. 3:**
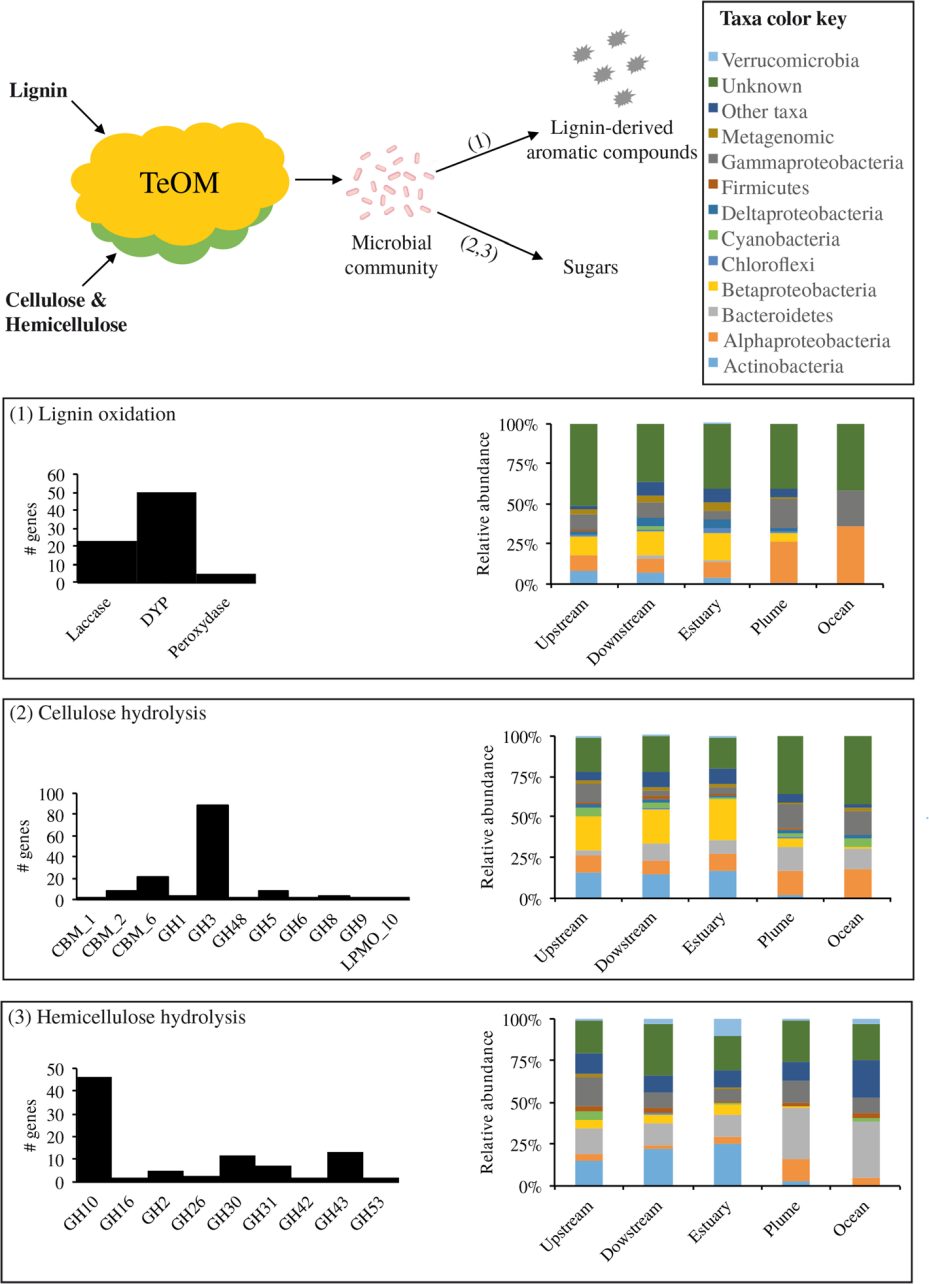
Enzymes potentially involved in the initial steps of TeOM degradation in the Amazon River microbiome. Lignin oxidation (1), cellulose (2) and hemicellulose degradation (3): the number of genes per family is shown (# genes). Taxa distribution per river section is also indicated

**Fig. 4:**
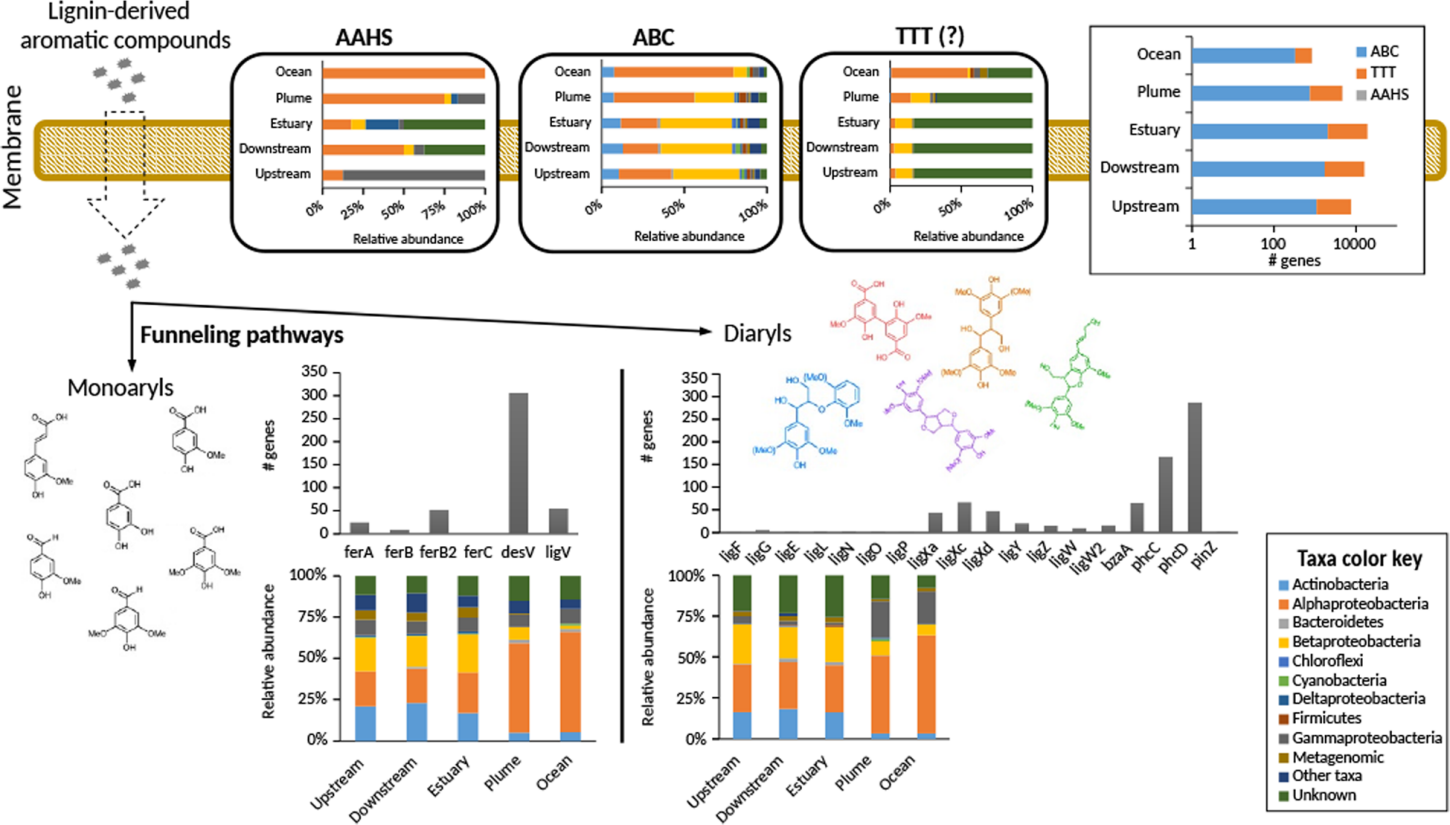
Transporters of lignin-derived compounds and funneling pathways of dimers and monomers in the Amazon River. The number of genes as well as taxonomy per protein family is indicated. The TTT system is depicted with (“?”) as its involvement is hypothetical. Funneling pathways to monomers and dimers are shown in terms of gene family and taxonomy

**Fig. 5:**
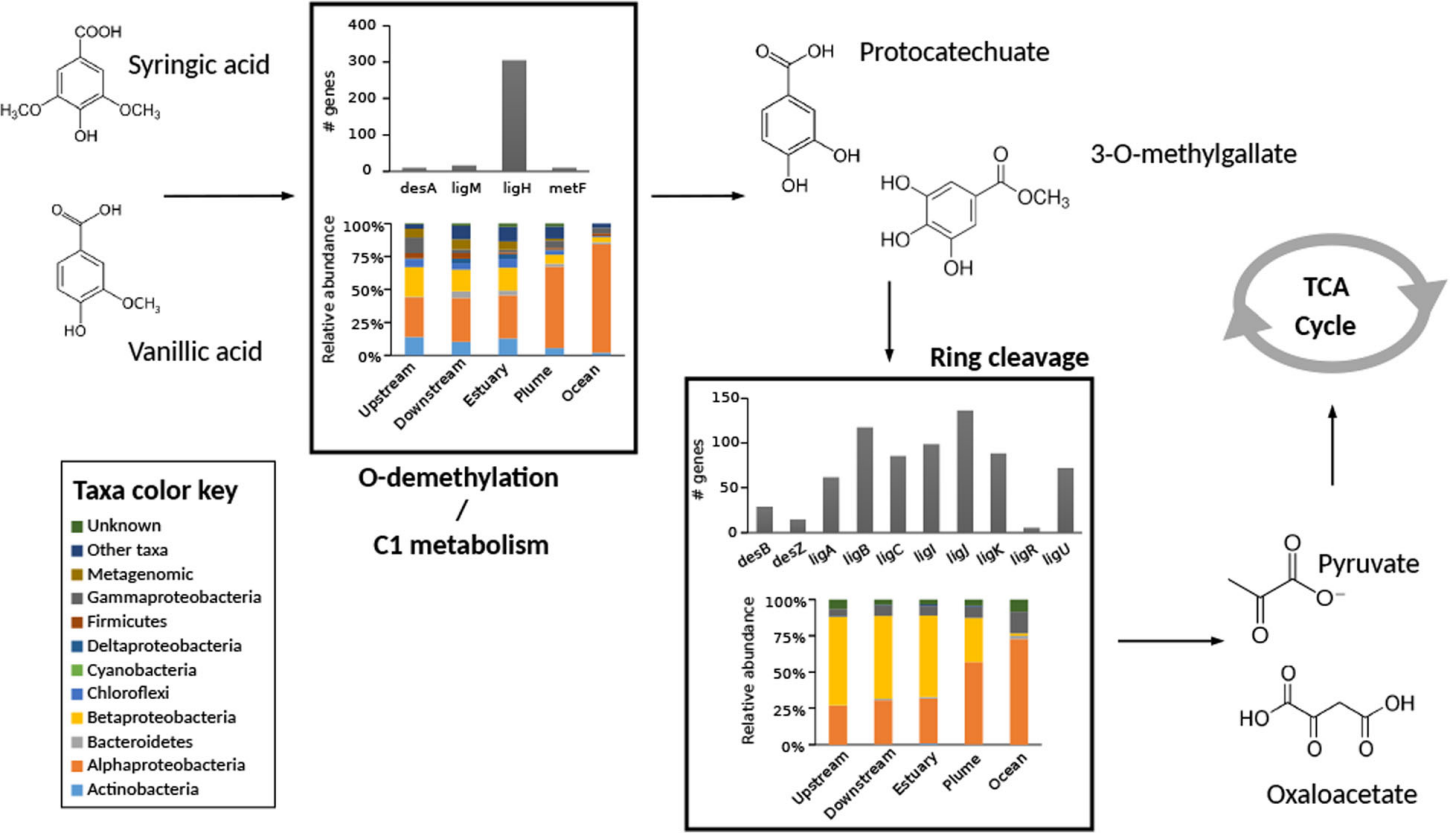
Main protein families potentially involved in the last steps of the lignin-derived compound metabolism. The O-demethylation and C1 metabolism of compounds is shown in terms of protein families and taxonomy (left), as well as the ring cleavage step that directs the substrates to pyruvate, which enters into the TCA cycle to be converted into ATP and CO_2_ (right)

A total of 6516 genes from the AMnrGC were identified as taking part in the potential TeOM degradation machinery from the Amazon River microbiome, being divided into cellulose degradation (143 genes), hemicellulose degradation (92 genes), lignin oxidation (73 genes), lignin-derived aromatic compounds transport and metabolism (2324 genes) and tricarboxylate transport (3884 genes) (Figs. 3, 4, and 5). The large number of gene variants associated with the metabolism of lignin-derived compounds and the transport of tricarboxylates (Fig. 4) reflects the variety of molecules generated during the lignin oxidation process in the Amazon River. No significant differences were found in the composition and distribution of genes in samples belonging to different microbial lifestyles (i.e. free-living vs. particle-attached). Eukaryotic contributions to the analysed functions were small (0.5–0.6%); thus, the machinery analysed hereafter is mostly prokaryotic.

### Lignin oxidation and deconstruction of cellulose and hemicellulose

TeOM consists of biopolymers, so the first step of its microbial-based degradation consists in converting polymers into monomers. Thus, the identified genes potentially involved in the oxidation of lignin and the degradation of cellulose and hemicellulose were investigated (Fig. 3). We observed a ubiquitous dominance of glycosyl hydrolase GH3, related to cellulose degradation. This function represented 63.2–65.3% of the genes possibly associated with this catabolic step across all river sections (71 ± 8 genes per section) (Fig. 3). In turn, hemicellulose degradation is potentially performed mostly by glycosyl hydrolase GH10 (52-56% of genes) in all river sections (35 ± 6 genes per section). Analysis of gene taxonomy (Fig. 3) indicated that cellulose and hemicellulose hydrolysis could be carried out predominantly by known taxa in fresh (70–81%) and brackish waters (58–79%). Cellulose degradation is likely performed by *Betaproteobacteria* and *Actinobacteria* in freshwaters, while *Bacteroidetes*, *Alphaproteobacteria* and *Gammaproteobacteria* possibly dominate this step in the ocean and plume sections (Fig. 3). A limited fraction of hemicellulose degradation seems to be associated to *Gamma-* and *Deltaproteobacteria*, which display a ubiquitous distribution along the river course. In turn, *Actinobacteria* and *Betaproteobacteria* (in freshwaters), and *Alphaproteobacteria* (in brackish waters) seem to contribute predominantly with functions related to hemicellulose degradation in different river sections (Fig. 3).

We found that lignin oxidation in the Amazon River may be mainly mediated by dye-decolorizing peroxidases (DyPs), as 61.5–71.2% of the genes potentially involved in this step were predominantly associated with freshwater areas. Only laccases (19 ± 2 genes per section) and peroxidases (42 ± 6 genes per section) were found in the Amazon River microbiome, no other families involved in lignin oxidation, like phenolic acid decarboxylase or glyoxal oxidase, were found (Fig. 3). Lignin oxidation appears to be encoded by genes belonging predominantly to taxonomically unassigned taxa (36–42%) in all river sections, as well as *Betaproteobacteria* in freshwaters and *Alpha-* / *Gammaproteobacteria* in brackish waters. Moreover, there is a possible redundancy of functions in *Actinobacteria* and *Alphaproteobacteria*, as they have contrasting abundances in fresh- and brackish waters.

### Lignin-derived compound transport

Lignin-derived aromatic compounds need to be transported from the extracellular environment to the cytoplasm prior to their degradation. Transporters that could be associated with lignin degradation (AAHS family and ABC transporters) were found in the AMnrGC (Fig. 4). Alphaproteobacterial AAHS and ABC genes had an increased abundance in plume and ocean samples (Fig. 4). Thus, *Alphaproteobacteria* contribute with their functional machinery to the metabolism of lignin-derived compounds in the Amazon River ecosystem. ABC transporters from *Betaproteobacteria* were enriched in freshwater or brackish river sections, while ABC transporters belonging to *Alphaproteobacteria* were enriched in sections with a higher salinity (Fig. 4). Different to the main taxa potentially involved in the degradation of TeOM, *Actinobacteria* had a smaller functional contribution to the transport of lignin-derived compounds, being more uniformly distributed along the river course (Fig. 4).

The tripartite tricarboxylate transporting (TTT) system is composed by three proteins, where *tctC* is responsible for capturing substrates in the extracellular space and bringing them to the transporting channel made by the proteins *tctA* and *tctB*, which recognize the substrate binding protein and move the substrate across membrane into the cytoplasm. The TTT system is suitable for transporting humic acids, and therefore lignin-derived compounds. There was a large number of gene variants (from 10 to 100) associated with the substrate binding proteins (*tctC*). As each protein is specific to one or a few substrates, possibly there is a huge variety of substrates in the Amazon River ecosystem. The extensive contribution of TTT system genes from unknown taxa reflects our limited understanding about it (Fig. 4). Similar to the other mentioned transporters, TTT transporters from *Alphaproteobacteria* were enriched in plume and ocean samples, while TTT transporters from *Betaproteobacteria* were enriched in freshwater or brackish sections of the river (Fig. 4).

### Degradation of lignin-derived aromatic compounds

Following the initial degradation of lignin, diverse aromatic compounds are released. These can be divided into aromatic monomers (monoaryls) or dimers (diaryls), which can be processed through several biochemical steps (also called funneling pathways) until being converted into vanilate or syringate. These compounds can be processed through the ring cleavage pathways to form pyruvate or oxaloacetate, which can be incorporated to the tricarboxylic acid cycle (TCA) of cells, generating energy. All known functions taking place in the metabolism of lignin-derived aromatic compounds were found in the AMnrGC, except the gene *ligD*, a Cα-dehydrogenase for αR-isomers of β-aryl ethers. The possible degradation pathway of lignin-derived compounds in the Amazon River (Figs. 4 and **5**) included 772 and 449 genes potentially belonging to funneling pathways of diaryls and monoaryls, respectively (Fig. 4). Examination of the pathways starting with vanilate and syringate revealed 1059 genes likely to be responsible for the ring-cleavage pathway. Almost 47% of all genes related to the degradation of lignin-derived compounds in the AMnrGC belonged to 4 gene families (*ligH*, *desV*, *phcD* or *phcC*). These genes represent the main steps of intracellular lignin metabolism, which are (1) funneling pathways leading to vanilate/syringate (Fig. 4), (2.1) O-demethylation/C1 metabolism and (2.2) ring cleavage (Fig. 5).

The previously mentioned taxonomic patterns in lignin oxidation as well as in the lignin-derived compound transport were also observed in the genes potentially related to the funneling pathways (Fig. 4) including the posterior steps (Fig. 5). There was an enrichment of *Alphaproteobacteria* functions in the river sections closer to the ocean, while the number of *Actinobacteria* genes decreased in those sections (Figs. 4 and **5**). We also observed a decrease in the relative functional contribution of *Betaproteobacteria*, and an enrichment of functions from *Gammaproteobacteria* with increasing salinity (Figs. 4 and 5).

### Potential to degrade TeOM among low-rank taxonomic levels

Even though higher taxonomic levels are informative on the main distribution trends of the TeOM degradation potential along the Amazon River, lower taxonomic levels can help linking functions with the actual species or genomes carrying them. Yet, accuracy tends to decrease as genes are taxonomically annotated at lower taxonomic ranks, given that many low-rank taxa are still missing or poorly represented in reference databases. Therefore, we used in most analyses high-rank taxonomic annotations, but we also investigated main trends in the distribution of low-rank taxa that may contribute genes to TeOM degradation in the Amazon River. Specifically, we analysed genes associated to TeOM degradation that could be taxonomically assigned to genera or genomes from unknown genera present in the Genome Taxonomy Database (GTDB) [42]. Only genera or genomes contributing functions in more than half of the samples in each of the river sections as well as in the plume and ocean samples were considered.

Our results point to a limited number of widespread genera or genomes contributing with their functional machineries to the TeOM degradation in the Amazon River system, especially in saline samples from the ocean and plume (Table 1). Mainly two low-rank taxa, HIMB11 (Rhodobacteraceae) and *Pelagibacter*, contributed functions to all TeOM-degradation steps in ocean and plume samples, except for the hydrolysis of cellulose (Table 1). In turn, low-rank taxa contributing genes to TeOM degradation in the Amazon River sections were more diverse than those present in ocean and plume samples, suggesting the existence microbial consortia (Table 1). Genera such as *Ramlibacter*, *Planktophila, Methylopumilus*, *Limnohabitans* and *Polynucleobacter* were enriched in TeOM degradation pathways along the Amazon River (Table 1). Overall, there was a clear salinity divide in terms of the main genomes or genera carrying out TeOM degradation in the Amazon River and in the plume and ocean areas.

**Table 1:** Low-rank taxa contributing genes to TeOM degradation in the Amazon River system

Zone	Genera or closest reference genomes from GTDB^a^	TeOM degradation step
**Upstream, downstream, and estuary**	*Ramlibacter*	Lignin oxidation
	*Planktophila*	Hemicellulose hydrolysis
	*Methylopumilus*, *Planktophila*, *Polynucleobacter*	Cellulose hydrolysis
	*Acidovorax*_D, *Cupriavidus*, *Curvibacter*_A, *Fonsibacter*, *Hylemonella*, *Ideonella*_A, *Limnohabitans*, PALSA-911 (Acetobacteraceae), *Polaromonas*, *Polynucleobacter*, *Ramlibacter*, *Reyranella*, SCGC-AAA027-K21 (Burkholderiaceae), UBA3064 (Burkholderiaceae), UBA6679 (Burkholderiaceae), Z2-YC6860 (Xanthobacteraceae)	TTT system
	AAA044-D11 (Nanopelagicaceae), AcAMD-5 (Nanopelagicales), GCA-2737595 (Nanopelagicaceae), *Limnohabitans*, *Nanopelagicus*, *Planktophila*, *Polynucleobacter*, RS62 (Burkholderiaceae), UBA6679 (Burkholderiaceae), UBA7398 (Nanopelagicaceae)	ABC transporters
	*Planktophila*, *Polynucleobacter*	FP-dimers
	*Limnohabitans*	FP-monomers
	GCA-2737595 (Nanopelagicaceae), *Methylopumilus*, *Planktophila*, *Fonsibacter*	O-demethylation/C1 metabolism
	*Acidovorax*_D, *Curvibacter*_A, *Limnohabitans*, *Pelomonas*	Ring cleavage
**Ocean and plume**	HIMB11 (Rhodobacteraceae), *Pelagibacter*	Lignin oxidation
	HIMB11 (Rhodobacteraceae)	Hemicellulose hydrolysis
	D2472 (Gammaproteobacteria), UBA4465 (Cyclobacteriaceae)	Cellulose hydrolysis
	HIMB11 (Rhodobacteraceae), HIMB59 (Alphaproteobacteria), *Pelagibacter*	TTT system
	*Pelagibacte*r, *Pelagibacter*_A, TMED189 (Acidimicrobiia)	ABC transporters
	HIMB11 (Rhodobacteraceae), *Pelagibacter*	FP-dimers
	HIMB11 (Rhodobacteraceae), *Pelagibacter*	FP-monomers
	HIMB11 (Rhodobacteraceae), *Pelagibacter*, SCGC-AAA076-P13 (Gammaproteobacteria)	O-demethylation
	N/A	Ring cleavage

Main prokaryotic genera, or genomes from the Genome Taxonomy Database (GTDB) without assigned genera, contributing genes to TeOM degradation in the Amazon River sections as well as in plume and ocean samples are indicated. Only taxa contributing functions in more than half of the samples of each studied zone are reported

^a^Genera or GTDB reference-genome names are indicated. For reference genome names, the lowest taxonomic level indicated in GTDB is shown in brackets

*FP* funneling pathways, *TTT* tripartite tricarboxylic transporter, *N/A* not applicable

### Spatial distributions

**Fig. 6:**
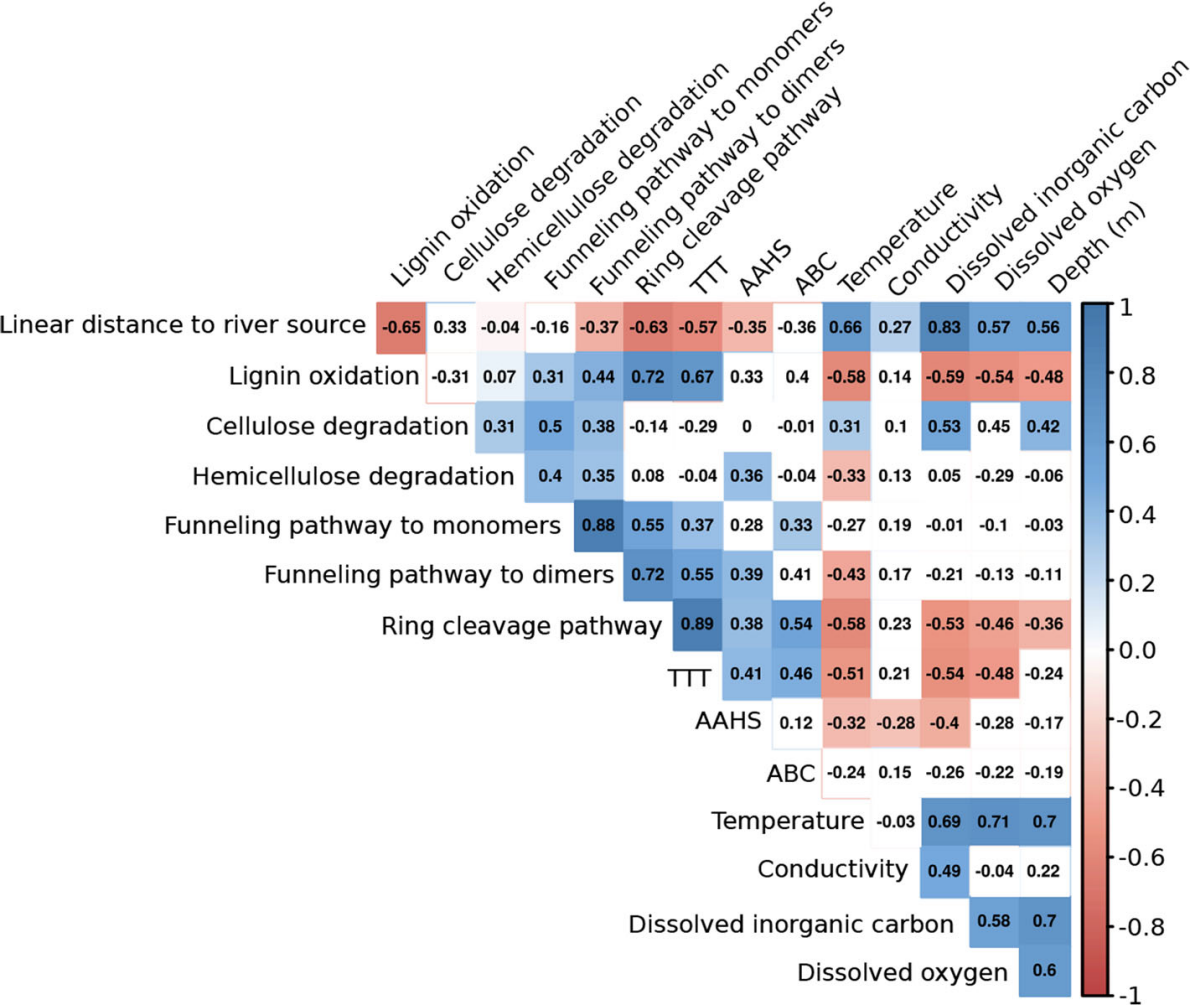
Correlations among genes associated with the processing of TeOM and their correlation to environmental variables. Correlations between the number of genes associated with lignin oxidation, cellulose and hemicellulose deconstruction, transporting systems (AAHS, ABC and TTT), lignin-derived aromatic compounds processing pathways (Ring cleavage pathways; Funneling pathways of dimers and monomers), and environmental variables (dissolved inorganic carbon—DIC, dissolved oxygen—DO, temperature, conductivity, sample depth—Depth and linear distance from the sampling site to the Amazon River source). Correlation coefficients are shown inside the boxes, and their color indicates the correlation strength. White boxes are non-significant correlations (*p* > 0.01)

We evaluated whether genes potentially associated with TeOM degradation displayed spatial distribution patterns along the river course (Fig. 6; Supplementary Table 4 in Additional file 1). For this, we used the linear geographic distance of sampling sites to the Amazon River source in Peru. The linear distance to the river source was negatively correlated with the number of genes possibly associated with lignin oxidation (*R*_Pearson’s_ = − 0.65, p-val. = 7.3 × 10^−11^), ring cleavage pathway (*R*_Pearson’s_ = − 0.63, p-val. = 1.2 × 10^−11^), tripartite tricarboxylate transporting (*R*_Pearson’s_ = − 0.57, p-val. = 5.4 × 10^−10^) and the AAHS transporters (*R*_Pearson’s_ = − 0.35, p-val. = 4.5 × 10^−6^) (Fig. 6**)**. This is coherent with a putative trend displayed by gene-function distributions along the river, pointing to lignin oxidation-related functions being replaced by cellulose degradation counterparts in brackish waters. A potential reduction of the microbial gene repertoire related to lignin processing as the river approaches the ocean suggests the ageing of TeOM during its flow through the Amazon River.

AAHS transporters were negatively correlated to the distance to the Amazon River source (*R*_Pearson’s_ = − 0.35, p-val. = 4.5 × 10^−6^), while ABC transporters were not correlated with this distance (*p* > 0.01) (Fig. 6). Furthermore, AAHS and ABC transporters showed positive correlations to the funneling pathway of diaryls (*R*_Pearson’s_ = 0.39, p-val. = 2.5 × 10^−3^) and monoaryls (*R*_Pearson’s_ = 0.33, p-val. = 9.6 × 10^−3^), respectively. This suggests specificity in the transport of lignin-derived molecules by those transporter families. Furthermore, AAHS (*R*_Pearson’s_ = 0.38, p-val. = 2.2 × 10^−5^) and ABC (*R*_Pearson’s_ = 0.54, p-val. = 6.7 × 10^−5^) transporters were positively correlated to the ring cleavage pathway, suggesting that ABC and AAHS transporters are relevant for the metabolism of lignin-derived compounds.

The number of genes in the TTT system displayed a negative correlation with the distance to the Amazon River source (R_Pearson’s_ = − 0.57, p-val. = 5.4 × 10^−10^), suggesting their predominance in freshwater sections of the river (Fig. 6). TTT transporters showed a positive correlation with lignin oxidation genes (R_Pearson’s_ = 0.67, p-val. = 6.7 × 10^−14^), suggesting they could be transporting lignin-derived products or a TTT coupling with the machinery to oxidize lignin. The TTT system was positively correlated to AAHS (*R*_Pearson’s_ = 0.41, p-val. = 3.5 × 10^−5^) and ABC (*R*_Pearson’s_ = 0.46, p-val. = 2 × 10^−4^) transporters (Fig. 6) pointing to a possible functional complementarity, as the TTT would transport substrates not transported by the other transporter families.

The gene machinery associated with the processing of lignin-derived aromatic compounds was positively correlated to the machinery related to lignin oxidation along the river course (Fig. 6), suggesting a co-processing of lignin and its byproducts. In terms of genes, cellulose degradation was not correlated with lignin oxidation (*p* > 0.01), but had a modest positive correlation to hemicellulose degradation (*R*_Pearson’s_ = 0.31, p-val. = 5.4 × 10^−3^) (Fig. 6), suggesting a coupling between both pathways.

We found correlations between both genes associated with the funneling pathway of dimers (*R*_Pearson’s_ = − 0.37, p-val. = 8 × 10^−5^) and the ring cleavage pathway (*R*_Pearson’s_ = − 0.63, p-val. = 1.2 × 10^−11^), with the distance to the Amazon River source (Fig. 6). This indicates that the degradation of lignin-derived aromatic compounds may follow a similar pattern as the lignin oxidation machinery, being predominantly restricted to upstream sections of the river. Moreover, the number of genes potentially related to hemi-/cellulose degradation was positively correlated to those possibly related to funneling pathways of lignin-derived monomers and dimers. This could reflect a potential co-metabolism of lignin-derived compounds and hemi-/cellulose degradation, instead of lignin oxidation.

### The effect of environmental variables in the potential TeOM degradation machinery

The potential processes related to TeOM degradation also seem to be correlated to specific environmental variables (Fig. 6; Supplementary Table 4 in Additional file 1). The machinery related to the oxidation of lignin is potentially more abundant in river sections with lower temperatures (*R*_Pearson’s_ = − 0.58, p-val. = 2 × 10^−4^), lower dissolved inorganic carbon (DIC) (*R*_Pearson’s_ = -0.59, p-val. = 1 × 10^−9^) and oxygen (DO) (*R*_Pearson’s_ = − 0.54, p-val. = 2 × 10^−4^), and at smaller depths (*R*_Pearson’s_ = − 0.48, p-val. = 1 × 10^−3^). Similarly, the hemicellulose degradation machinery was negatively correlated with temperature (*R*_Pearson’s_ = − 0.33, p-val. = 9 × 10^−4^). In contrast to those mentioned above, the cellulose degradation arsenal was positively correlated to higher temperatures (*R*_Pearson’s_ = 0.31, p-val. = 8 × 10^−4^), DIC (*R*_Pearson’s_ = 0.53, p-val. = 1 × 10^−7^) and sampling depth (*R*_Pearson’s_ = 0.42, p-val. = 5 × 10^−5^).

The transporters of lignin-derived molecules were marginally correlated to the measured environmental variables (Fig. 6). Specifically, ABC transporters did not correlate to any variable; however, AAHS transporters were negatively correlated to temperature (*R*_Pearson’s_ = − 0.32, p-val. = 6 × 10^−3^), DIC (*R*_Pearson’s_ = − 0.40, p-val. = 1 × 10^−4^) and conductivity (*R*_Pearson’s_ = − 0.28, p-val. = 3 × 10^−3^). TTT transporters seemed to follow a similar trend as lignin oxidation in terms of temperature (*R*_Pearson’s_ = − 0.51, p-val. = 7 × 10^−7^), DIC (*R*_Pearson’s_ = − 0.53, p-val. = 8 × 10^−8^) and DO (*R*_Pearson’s_ = − 0.48, p-val. = 2 × 10^−5^).

The initial steps of the metabolism of lignin-derived molecules did not seem to be correlated to environmental heterogeneity (Fig. 6), except for the functions related to the funneling pathways of dimers that were correlated to temperature (*R*_Pearson’s_ = − 0.43, p-val. = 5 × 10^−6^). The last steps of the processing of lignin-derived molecules, the ring cleavage, were strongly correlated to environmental heterogeneity. These final steps resembled patterns observed in the lignin oxidation machinery in terms of temperature (*R*_Pearson’s_ = − 0.58, p-val. = 4 × 10^−8^), DIC (*R*_Pearson’s_ = − 0.53, p-val. = 1 × 10^−7^), DO (*R*_Pearson’s_ = − 0.46, p-val. = 4 × 10^−5^) and sampling depth (*R*_Pearson’s_ = − 0.36, p-val. = 4 × 10^−3^).

## Discussion

The AMnrGC significantly expands the comprehension of the metabolic potential of the world’s largest river microbiome and is publicly available (10.5281/zenodo.1484504). The predicted ~ 3.7 M genes are a valuable resource for understanding the functioning of this ecosystem as well as for bioprospecting. Almost half of the genes in the catalogue had no close orthologs, suggesting gene novelty. Yet, this extensive portion of unknown genes (48%) is similar to other environmental microbiomes that featured 40–60% of unknown genes [6–8]. Interestingly, the analysis of k-mers indicated a distinct composition, in terms of genomic information, of the Amazon River microbiome when compared to other rivers and to the Amazon rainforest soil, being coherent with the novel diversity previously found in Brazilian freshwater systems [43]. Altogether, this points to gene novelty and a compositional distinctiveness of the Amazon River microbiome.

Analyses of COG functions pointed to a number of core functions along the Amazon River course, which was supported by the similar distribution of COG superclasses along the different river sections (Fig. 2d). In particular, COG functions within the superclass “Metabolism” were the most abundant in the AMnrGC, as well as in the upper Mississippi River [44]. Core functions included a general carbohydrate metabolism and several transporter systems, mainly ABC transporters. This suggests a sophisticated machinery to process TeOM in the Amazon River, with core metabolisms indicating a general organic matter degradation system, ending in acetogenic pathways.

Lignin-derived aromatic compounds need to be transported from the extracellular milieu to the cytoplasm to be degraded, and different transporting systems can be involved in this process [36, 37, 45, 46]. In particular, previous studies showed that the TTT system was present in high quantities in the Amazon River, and this was attributed to a potential degradation of allochthonous organic matter [14]. Recent findings also suggest a TTT system related to the transport of TeOM degradation byproducts [47, 48]. Little is known about these transporters, but our findings indicate that TTT is an abundant protein family in the Amazon River, suggesting that tricarboxylates are a common carbon source for prokaryotes in these waters. Our results also indicated that the TTT transporters could be linked to the genes potentially related to lignin oxidation, supporting the role of TTT in TeOM degradation.

The taxa found to be potentially involved in TeOM degradation mostly belong to *Proteobacteria* (Table 1**)**, especially *Betaproteobacteria* (such as the genera *Polynucleobacter*, *Methylopumilus* and *Limnohabitans*) and *Alphaproteobacteria* (e.g. HIMB11 and *Candidatus* Pelagibacter). Other important groups include *Actinobacteria* (represented by the genus *Candidatus* Planktophila) and *Bacteroidetes*, all regular freshwater taxa [49]. The participation of *Bacteroidetes* in hemi-/cellulose degradation has been previously reported, being metabolically capable to degrade recalcitrant organic compounds such as humic substances [49, 50]. In the Amazon River, there was an increase of *Gammaproteobacteria* and *Alphaproteobacteria* genes, possibly involved in TeOM degradation, in the river sections closer to the ocean. In turn, there was an increase in the number of TeOM degradation genes from *Actinobacteria* and *Betaproteobacteria* in freshwater sections of the Amazon River. This suggests that salinity shapes the composition of microbial communities in the different sections of the Amazon River and consequently affects their ecology, agreeing with the salinity boundary hypothesis introduced by Logares et al. [51]. The distribution of bacterial taxa along the Amazon River may also reflect taxon-specific preferences for TeOM quality, as previous studies have reported a differential preference of bacteria for fresh vs. aged TeOM [52]. For example, after long incubation experiments, *Actinobacteria*, *Bacteroidetes* and *Betaproteobacteria* showed a preference for fresh TeOM while *Alphaproteobacteria* and *Gammaproteobacteria* displayed a preference for aged organic matter. Furthermore, Sipler et al. [53] found that Arctic coastal *Alphaproteobacteria*, *Bacteroidetes*, *Betaproteobacteria* and *Actinobacteria* were negatively affected by the addition of TeOM in bottle experiments. This suggests that most TeOM degradation occurs in rivers and that coastal microbiomes are less capable to degrade these compounds, being coherent with our results.

Correlations between measured environmental variables and the potential TeOM degradation machinery indicated that lignin oxidation may happen in regions with low concentration of oxygen and dissolved inorganic carbon, and low temperature. This is coherent with previous reports [38–41]. However, the machinery related to the degradation of lignin-derived compounds seems to be independent of environmental conditions, indicating a potentially ubiquitous degradation of such compounds in the Amazon River. The correlations between environmental variables and the potential degradation of cellulose and hemicellulose still suggest that cellulose and lignin would be degraded in different river sections. Therefore, both processes seem to be decoupled in the Amazon River.

**Fig. 7:**
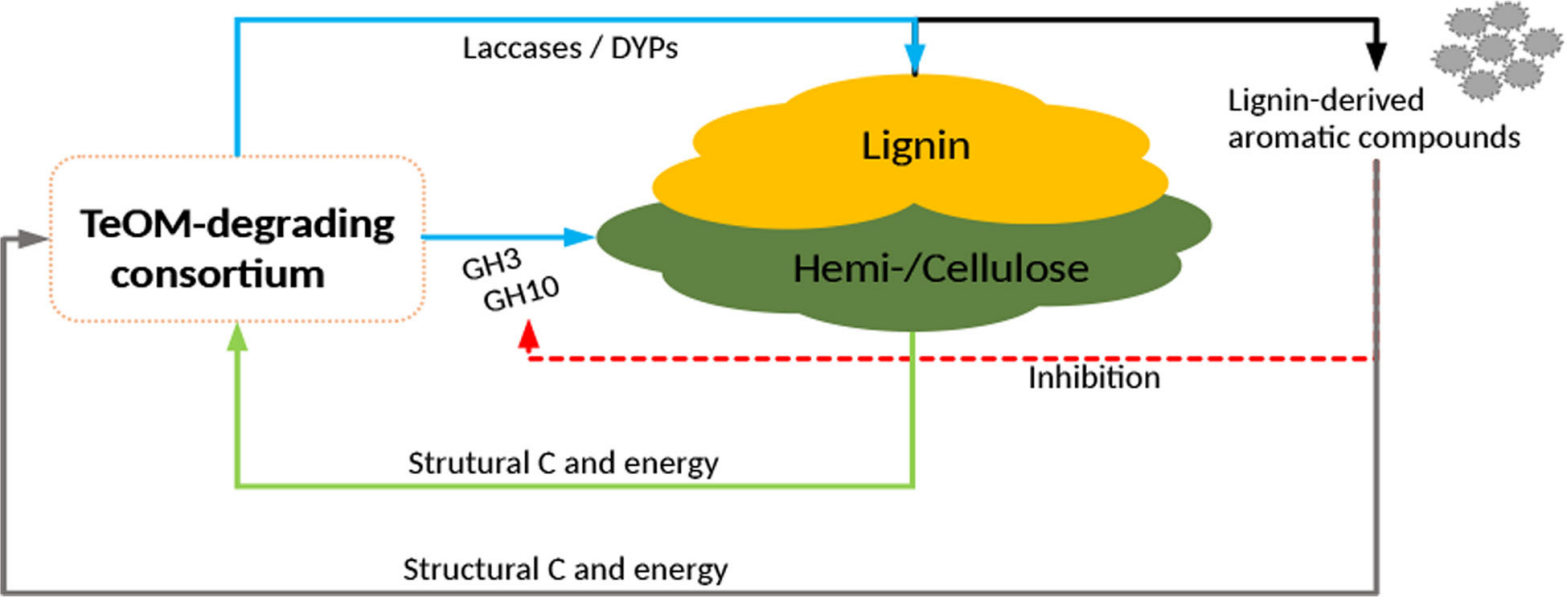
*Priming effect* model of microbial TeOM degradation in the Amazon River. The cellulolytic communities degrade hemi-/cellulose through secretion of glycosyl hydrolases (mainly GH3/GH10), which release sugars to the environment. These sugars can promote growth of the cellulolytic and lignolytic communities, and during this process, the oxidative metabolism produces reactive oxygen species (ROS). ROS activate the exoenzymes (mainly DYPs and laccases) secreted by the lignolytic community to oxidize lignin. After lignin oxidation, the hemi-/cellulose becomes exposed again, helping the cellulolytic communities to degrade it. During the previous process, several aromatic compounds are formed, which can potentially inhibit cellulolytic enzymes and microbial growth. However, these compounds are consumed by lignolytic microorganisms, reducing their concentration in the environment allowing decomposition to proceed

Our results agree with previous experimental evidence of TeOM ageing in the Amazon River [27, 28], which supported a *priming effect* in incubation experiments with recalcitrant and labile organic matter [20]. However, the mechanism behind this *priming effect* remained unexplained. Based on our findings, we hypothesized a model for the potential *priming effect* acting in lignocellulose complexes in the Amazon River (Fig. 7). In this model, there are two different communities co-existing in a consortium: one possibly responsible for hemi-/cellulose degradation and another one likely involved in lignin degradation. The first community would release extracellular enzymes (mainly glycosyl hydrolases from families GH3 and GH10), producing different kinds of carbohydrates. These carbohydrates may provide structural carbon and energy for the entire consortium. The potential lignolytic community would also use the cellulolytic byproducts to grow, which promotes an oxidative metabolism. This oxidative metabolism could trigger the production and secretion of reactive oxygen species (ROS) (Fig. 7). ROS are then used by DyPs and laccases secreted by these putative lignolytic communities to oxidize lignin, exposing more hemi-/cellulose to cellulolytic communities, re-starting the cycle (Fig. 7). Another important role of lignolytic communities could be the degradation of lignin-derived aromatic compounds generated by the lignin oxidation process. Those compounds, if not degraded, can inhibit cellulolytic enzymes and microbial growth [54–57], preventing TeOM degradation. This cycle may be considered as a *priming effect*, where both communities benefit from each other.

## Conclusions

The Amazon River is a major carbon link between terrestrial, atmospheric and marine ecosystems. Our work represents a first effort to link the TeOM inputs into the Amazon River with the microbial metabolisms potentially responsible for their degradation. We identified genes and metabolisms that are likely key in TeOM degradation. Furthermore, our results indicate differential distributions of TeOM-related genes that in some cases seem to be driven by environmental heterogeneity. Our work also generated the AMnrGC, an important resource for interrogating the functionality of the Amazon River microbiome as well as for bioprospecting. Given the extent and difficulty of access to many regions of the Amazon River basin, our work is an important first step that could pave the road for future ambitious sampling campaigns that will investigate gene expression, metaproteomics and the capacity of the Amazon River microbiota to degrade TeOM in field or laboratory experiments.

## Materials and methods

We analysed 106 metagenomes [12, 13, 15, 18] from 30 sampling points distributed along the Amazon river basin, with an average coverage of 5.3 × 10^9^ (± 7.4 × 10^9^) base pairs per metagenome (Supplementary Table 1 in Additional file 1). The sampling points from the Solimões River and lakes in the Amazon River course, located upstream from the city of Manaus, until the Amazon River’s plume in the Atlantic Ocean covered ~ 2106 km and were divided into 5 sections (Fig. 1a and Supplementary Table 1 in Additional file 1). These sections were (1) *upstream section* (upstream Manaus city), (2) *downstream section* (placed between Manaus and the start of the Amazon River estuary. It includes the influx of particle-rich white waters from the Solimões River as well as the influx of humic waters from Negro River [58, 59]), (3) *estuary section* (part of the river that meets the Atlantic Ocean), (4) *plume section* (the area where the Ocean is influenced by the Amazon River inputs) and (5) *ocean* (the area with higher salinity surrounding the Plume).

Samples were taken as previously indicated [12, 13, 15, 18]. Depending on the original study, particle-associated microbes were defined as those passing the filter of 300 μm mesh size and being retained in the filter of 2–5 μm mesh size. Free-living microbes were defined as those passing the filter of 2–5 μm mesh size, being retained in the filter of 0.2 μm mesh size. DNA was extracted from the filters as indicated in the original studies [12, 13, 15, 18]. Metagenomes were obtained from libraries prepared with either Nextera or TruSeq kits. Different *Illumina* sequencing platforms were used: Genome Analyzer IIx, HiSeq 2500 or MiSeq. Additional information is provided in Supplementary Table 1 in Additional file 1.

### Metagenome analysis

*Illumina* adapters and poor-quality bases were removed from metagenomes using Cutadapt [60]. Only reads longer than 80 bp, containing bases with Q ≥ 24, were kept. The quality of the reads was checked with FASTQC [61]. Reads from metagenomes belonging to the same sampling points were assembled together using MEGAHIT (v1.0) [62], with the meta-large presets. Only contigs > 1 kbp were considered, as recommended by previous work [63]. Assembly quality was assessed with QUAST [64]. Metagenome assembly yielded 2,747,383 contigs ≥ 1000 base pairs, in a total assembly length of ~ 5.5 × 10^9^ bp with an average N50 of 2064 ± 377 bp (see Supplementary Table 2 in Additional file 1).

### Analysis of k-mer diversity over different river zones

A k-mer diversity analysis was used to compare the genetic information of the Amazon River microbiome against that in other microbiomes from Amazon rainforest soil and temperate rivers (Supplementary Table 3 in Additional file 1). Specifically, the Amazon River metagenomes (106) were compared against 37 metagenomes from the Mississippi River [65], 91 metagenomes from three watersheds in Canada [66] and 7 metagenomes from the Amazon forest soil [67]. The rationale to include soil metagenomes was to check whether genomic information in the river could be derived from soil microbiota. K-mer comparisons were run with SIMKA (version 1.4) [68] normalizing by sample size. Low complexity reads and k-mers (Shannon index < 1.5) were discarded before SIMKA analyses. The resulting Jaccard’s distance matrix was used to generate a non-metric multidimensional scaling (NMDS) analysis. Permutation tests were used to check the homogeneity of *β* dispersion in the groups, and permutational multivariate analysis of variance (PERMANOVA/ANOSIM) was used to test the groups’ difference. Both analyses were performed using the R package Vegan [69].

### Amazon River basin Microbial non-redundant Gene Catalogue (AMnrGC)

Genes were predicted using Prodigal (version 2.6.3) [70]. Only open reading frames (ORFs) predicted as complete, accepting alternative initiation codons, and longer than 150 bp, were considered in downstream analyses. Gene sequences were clustered into a non-redundant gene catalogue using CD-HIT-EST (version 4.6) [71, 72] at 95% of nucleotide identity and 90% of overlap of the shorter gene [5]. Representative gene sequences were used in downstream analyses. GC content per gene was inferred via Infoseq, EMBOSS package (version 6.6.0.0) [73].

### Gene abundance estimation

The quality-checked sequencing reads were backmapped against our non-redundant gene catalogue using BWA (version 0.7.12-r1039) [74] and SamTools (version 1.3.1) [75]. Gene abundances were estimated using the software eXpress (version 1.5.1) [76], with no bias correction, as counts per million (CPM). We used a CPM ≥ 1.00 for a gene to be present in a sample, and an average abundance higher than zero (μ_CPM_ > 0.0) for a gene to be present in a river section or water type (i.e. freshwater, brackish water or the mix of them in the plume).

### Functional annotation

Representative genes (and their predicted amino acid sequences) were annotated by searching them against KEGG (Release 2015-10-12) [77], COG (Release 2014) [78], CAMERA Prokaryotic Proteins Database (Release 2014) [79] and UniProtKB (Release 2016-08) [80] via the Blastp algorithm implemented in Diamond (v.0.9.22) [81], with a query coverage ≥ 50%, identity ≥ 45%, e-value ≤ 1e^−5^ and score ≥ 50. Hits were parsed by score, e-value and identity until the best result was found. KO-pathway mapping was performed using KEGG mapper [82]. HMMSearch (version 3.1b1) [83] was used to search proteins against dbCAN (version 5) [84], PFAM (version 30) [85] and eggNOG (version 4.5) [86] databases, using an e-value ≤ 1e^−5^, and posterior probability of aligned residues ≥ 0.9, and no domain overlapping. Accumulation curves were obtained using random progressive nested comparisons with 100 pseudo-replicates for genes and PFAM predictions.

### Core metabolisms

We adopted the definition of core metabolic functions as those involved in cell or ecosystem homeostasis, representing the minimal metabolic machinery needed to survive in a given environment. Similar to other works [6, 7], we used the annotations with KEGG and PFAM databases to determine the bacterial functional core. By using gene abundances as CPM as a criterion for counting functions in each sample or river section, we analysed metabolic pathways. Those functions present in at least 80% of the samples were considered as core. KEGG Mapper [87] and MinPath [88] were used to organize the information underlying core functions.

### Gene taxonomy

Given that a high number of low-rank taxa are missing or poorly represented in reference databases, taxonomic annotation accuracy tends to decrease as genes are taxonomically annotated at lower taxonomic ranks. For this reason, we used two different approaches to taxonomically annotate genes. *Approach 1* is more conservative, aiming to annotate genes at higher taxonomic ranks (e.g. Class) and therefore being potentially more accurate than the less conservative *Approach 2*, which aims at annotating genes at lower taxonomic ranks (e.g. Genus). The specific methods associated to each approach are indicated below:

- *Approach 1:* High-rank gene taxonomy was assigned considering the best hits (score, e-value and identity; see above) using KEGG (Release 2015-10-12) [77], UniProtKB (Release 2016-08) [80] and CAMERA Prokaryotic Proteins Database (Release 2014) [79]. Taxonomic last common ancestors (LCA) were determined from TaxIDs (NCBI) associated with UniRef100 and KO entries. Information from the CAMERA database was also used to retrieve taxonomy (NCBI TaxID). Taxonomy was assigned using the best hit, of a given protein, obtained across databases. Proteins were annotated as “unassigned” if their taxonomic signatures were mixed, containing representatives from several domains of life, or if they had the function assigned without taxonomic information. Reference sequences with hits to poorly annotated sequences from other metagenomes were referred to as “Metagenomic”.
- *Approach 2:* Low-rank taxonomic affiliation was determined using MMseqs2 version 11-e1a1c [89] using default settings, based on the Genome Taxonomy Database (GTDB; publicly available in https://gtdb.ecogenomic.org/) [42].

### Potential TeOM degradation machinery

To investigate the potential TeOM degradation, we grouped samples by river section and assessed their gene content. Genes were then searched against reference sequences and protein families known to be involved in TeOM degradation (see Supplementary Table 5 in Additional file 1). In particular, bacterial lignin degradation starts with extracellular polymer oxidation followed by monomers and dimers moving across membranes into the cytoplasm for their ultimate degradation. Protein families related to lignin oxidation (PF05870, PF07250, PF11895, PF04261 and PF02578) were searched among PFAM-annotated genes. The genes related to the metabolism of lignin-derived aromatic compounds were annotated with Diamond (Blastp search mode; v.0.9.22) [81], with query coverage ≥ 50%, protein identity ≥ 40% and e-value ≤ 1e^−5^ as recommended by Kamimura et al. [45], using their dataset as reference.

Cellulose and hemicellulose degradation involve glycosyl hydrolases (GH). The most common cellulolytic protein families (GH1, GH3, GH5, GH6, GH8, GH9, GH12, GH45, GH48, GH51 and GH74) [90] and cellulose-binding motifs (CBM1, CBM2, CBM3, CBM6, CBM8, CBM30 and CBM44) [90, 91] were searched in PFAM/dbCAN annotations. In addition, the most common hemicellulolytic families (GH2, GH10, GH11, GH16, GH26, GH30, GH31, GH39, GH42, GH43 and GH53) [91] were searched in the PFAM/dbCAN database. Lytic polysaccharide monooxygenases (LPMO) [91] were also identified using PFAM to investigate the simultaneous deconstruction of cellulose and hemicellulose.

During the degradation of refractory and labile material by exoenzymes, microbes produce a complex mix of particulate and dissolved organic carbon. The use of this mix is mediated by a vast variety of transporter systems [46]. The typical transporters associated with lignin degradation (AAHS family, ABC transporters, MHS family, ITS superfamily and TRAP transporter) were searched with Diamond (v.0.9.22) [81], using query coverage ≥ 50%, protein identity ≥ 40% and e-value ≤ 1e^−5^ and a reference dataset previously compiled [45].

Similarly to the fate of hemi-/cellulose degradation byproducts, lignin degradation ends up in the production of 4-carboxy-4hydroxy-2-oxoadipate, which is converted into pyruvate or oxaloacetate, both substrates of the tricarboxylic acid cycle (TCA) [45]. Recently, several substrate binding proteins (TctC) belonging to the tripartite tricarboxylate transporter (TTT) system were associated with the transport of TeOM degradation byproducts, like adipate [47] and terephthalate [48]. To investigate the metabolism of these compounds, and the possible link between the TTT system and lignin/cellulose degradation, the protein families TctA (PF01970), TctB (PF07331) and TctC (PF03401) were searched in PFAM.

The genes found using the abovementioned strategy were submitted to PSORT v.3.0 [92], to determine the protein subcellular localization (cytoplasm, secreted to the outside, inner membrane, periplasm or outer membrane). We carried out predictions in the three possible taxa (Gram negative, Gram positive and Archaea), and the best score was used to determine the subcellular localization. Genes assigned to an “unknown” location, as well as those with a wrong assignment, were eliminated (for example, genes known to work in extracellular space that were assigned to the cytoplasmic membrane).

The total amount of TeOM degradation genes found per function (lignin oxidation, transport, hemi-/cellulose degradation and lignin-derived aromatic compounds metabolism) in each section of the river were normalized by the maximum gene counts per metagenome. Subsequently, correlograms were produced adding the environmental variables and using Pearson’s correlation coefficients calculated with complete pairwise observations using the R packages Corrplot [93] and RColorBrewer [94]. The linear geographic distance of each metagenome to the Amazon River source (i.e. Mantaro River, Peru, 10° 43′ 55″ S / 76° 38′ 52″ W) was also used in this analysis to infer changes in gene counts along the Amazon River course. The sampling site distance to the Amazon River source in Peru was calculated with the R package Fields [95].

## Supplementary information

**Additional file 1: Supplementary Tables.** (1) Description of the 106 metagenomes used to build the Amazon River basin Microbial non-redundant Gene Catalogue (AMnrGC). The Amazon River basin section shows the group that a sample belongs to according to its geographic location. Other features were obtained from the original publications and SRA codes. “N.A.” stands for not available. (2) Co-assembly groups used to build the Amazon River basin Microbial non-redundant Gene Catalogue (AMnrGC). (3) Metagenomes used for K-mer diversity assessment. (4) Correlation and significance between gene content and environmental variables. Pearson’s correlation coefficients are shown under the diagonal and correspondent p-values are shown in red above the diagonal. The correlations were calculated using complete pairwise observations. (5) Reference proteins and protein families involved in terrestrial organic matter degradation used to annotate proteins related to lignin oxidation, cellulose and hemicellulose degradation in the AMnrGC.

## Data Availability

Metagenomes used to construct the Amazon River gene catalogue (AMnrGC) are publicly available (See Supplementary Table 1 in Additional file 1) from the following SRA projects: SRP044326, PRJEB25171 and SRP039390). Publicly available metagenomes used in the k-mer diversity comparison are detailed in Supplementary Table 3 (Additional file 1) and publicly available from the following SRA projects: Amazon forest [PRJNA336764, PRJNA336766, PRJNA337825, PRJNA336700, PRJNA336765], Mississippi River [SRP018728] and Canada watersheds [PRJNA287840]). The AMnrGC and all the associated files are available in a permanent Zenodo repository (10.5281/zenodo.1484504).

## Abbreviations

AAHS: Aromatic Acid:H^+^ Symporter
ABC: ATP-binding cassette transporters
AMnrGC: Amazon River basin Microbial non-redundant Gene Catalogue
C1: One carbon compounds (e.g. methane)
COG: Clusters of Orthologous Genes
CPM: Counts per million
DIC: Dissolved inorganic carbon
DO: Dissolved oxygen
DyPs: Dye-decolorizing peroxidases
FP: Funneling pathway
GHn: Glycosyl hydrolase family “n”
KEGG: Kyoto Encyclopedia of Genes and Genomes
LCA: Taxonomic last common ancestors
LPMO: Lytic polysaccharide monooxygenases
MFS: Major facilitator superfamily of transporters
PFAM: Protein family
Pg C: Peta(10^15^) grams of carbon
p-val.: *p* value
ROS: Reactive oxygen species
TCA: Tricarboxylic acid cycle
Tct: Tripartite transporter component (A, B or C)
TeOM: Terrestrial organic matter
TTT: Tripartite tricarboxylate transporter/transporting system
